# Brick Debris Dust as an Ecological Filler and Its Effect on the Durability of Asphalt Mix

**DOI:** 10.3390/ma13215023

**Published:** 2020-11-07

**Authors:** Agnieszka Woszuk, Michał Wróbel, Lidia Bandura, Wojciech Franus

**Affiliations:** Department of Geotechnical Engineering, Faculty of Civil Engineering and Architecture, Lublin University of Technology, Nadbystrzycka 40, 20–618 Lublin, Poland; a.woszuk@pollub.pl (A.W.); m.wrobel@pollub.pl (M.W.); l.bandura@pollub.pl (L.B.)

**Keywords:** brick debris dust, mineral filler, asphalt mix, air voids, water sensitivity, stiffness modulus

## Abstract

Brick debris is one of the main construction wastes obtained from demolition of buildings. However, this material can be successfully used in the recycling process. The purpose of the study was to determine the brick dust addition effect on asphalt surface service life. An asphalt concrete reference mix was designed for bonding layer and prepared using a Marshall compacting device. In addition, three mixes with combined lime-brick filler were prepared as well as one mix containing only brick filler. The samples were tested for their volumetric properties—density, bulk density, air void content, resistance to water and frost and stiffness modulus with varying test temperatures. It was found that 25% of brick dust addition to the filler did not considerably change the properties of the tested samples, while in the case of 50% filler the replacement stiffness and frost resistance decrease; however, the minimum required value is maintained. It can be concluded that the lime filler can be replaced with up to 50% of brick dust without a negative impact on the properties of asphalt mix. The proposed solution fits into the idea of sustainable development indicating a way of brick debris management.

## 1. Introduction

A properly formed road network is an important factor in the economic development of each country. For this reason, in many regions, investment in construction of new roads and modernization of existing ones is being increased every year. However, both in Europe and the world, asphalt pavements prevail. In the United States, asphalt mix production increased by 7.8% between 2014 and 2017, while in Europe the value increased by 12.5% [1]. At the same time, the development of road infrastructure is associated with significant consumption of natural resources, including mainly crude oil and mineral aggregates as well as pollution of the environment through the emission of hazardous compounds and the generation of waste materials [2]. Therefore, it is becoming increasingly crucial to use sustainable solutions, for instance warm mix asphalt technology, in the road construction industry [3,4,5]. Another way to achieve clean asphalt mix production is to use waste materials interchangeably as mineral aggregates [6,7] or as an additive affecting the properties of the asphalt binder [8,9]. Furthermore, the mineral filler, which is ground limestone in a conventional asphalt mix, can be replaced by waste material such as: lime kiln dust [10], waste glass powder [11], fly ash [12], ash from biomass combustion [13], cement and calcium hydroxide [14], waste oyster shells [15], calcium carbide [16] and others [17].

Brick debris is one of the main contributors to building waste. In some countries it represents up to 30% of this waste [18]. In recent years, there have been many studies on the reuse of brick debris. Padmini et al. evaluated the physical properties of brick debris aggregates in comparison to light natural aggregates [19]. Kibriya and Speare found that concrete with crushed bricks has similar compressive, tensile and flexural strengths to concrete with natural aggregates [20]. Poon et al. used 50% addition of crushed brick in the production of paving blocks [21,22]. Moreover, it is possible to use brick debris aggregate in unbound subbase materials as well as cement-stabilised base layers successfully [18,23]. Research studies of asphalt with the addition of ground brick debris were also conducted. Asphalt mastic containing recycled red brick powder at 0 °C was found to have higher shear creep stiffness modulus than mastic containing lime filler [24], which may cause low-temperature cracking. Also the results of the research carried out by Wu et al. indicate that the dust from brick debris can have a certain positive effect on the high-temperature performance and, at the same time, a negative effect on the low-temperature properties of asphalt mastic [25]. The tests performed on asphalt concrete asphalt mix type also proved to have higher resistance to permanent deformation with the use of brick dust filler [26]. On the other hand, porous asphalt with the addition of brick dust was characterized by a deeper rut compared to a mixture with lime filler [27]. Differences in test results may result from the use of different testing procedures as well as differences in the properties of individual materials.

The physicochemical characteristics are particularly important for a filler that performs numerous important functions in the asphalt mix [28,29]:

(1) fills the void spaces between mineral mix grains;
(2) stiffens the asphalt forming the mineral filled asphalt together with the binder which binds the aggregate mix grains;
(3) improves water tightness and frost resistance of an asphalt mix.

The chemical composition of brick debris dust differs significantly from the lime filler commonly used in mineral–asphalt mixtures, which may adversely affect the performance of the mixtures, especially their water resistance. At the same time, environmental aspects speak in favor of the use of waste materials in road construction. In the works published so far, either the influence of brick dust on the properties of asphalt mastic was determined or lime filler was replaced completely by brick dust. The analyses presented in the paper were performed for asphalt mix with brick and lime filler combination in 4 different proportions. The main aim of the study was to determine the brick dust addition effect on the asphalt surface service life defined as resistance to water and frost, considering physical and chemical properties of the brick dust.

## 2. Experimental Materials

The limestone used as the standard mineral filler was acquired from the Bukowa mine (Poland). Dust from brick debris was obtained by grinding brick waste obtained during demolition of old buildings in a laboratory jaw crusher LKS-100 (Testchem, Poland). The physical properties of these materials are summarized in Table 1.

The bitumen of 35/50 penetration grade was obtained from ORLEN Asfalt Sp. z o.o (Płock, Poland). The following tests were performed in order to characterize the basic bitumen properties: penetration, ring and ball softening point and viscosity. On the basis of the penetration and softening point test results, the penetration index defining the thermal vulnerability of asphalt was calculated. The test results are presented in Table 2.

**Figure 1:**
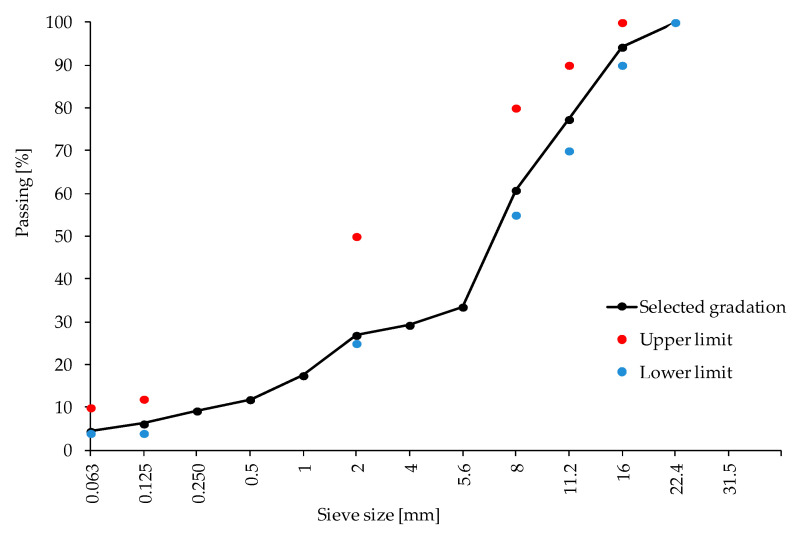
Aggregate gradation of the reference asphalt mix.

Particle size distribution of the aggregates was assessed using the dry sieving method (EN 933-1:2012), while the mineral filler particle size distribution was assessed using the air jet sieving method (EN 933-10:2009). The aggregate gradation of the reference mix asphalt is given in Figure 1. The mineral mix composition was designed using the grading envelope method. Sub-threshold values apply to technical requirements effective in Poland [30].

Asphalt concrete mixture for binder course AC 16 W was used as a reference material, abbreviated further as AC. The amount of asphalt added was 4.5% in relation to the asphalt mix weight. The designed mix meets the technical regulations in force in Poland [30]. Table 3 shows the composition of both the mineral mixture and the asphalt mix.

**Figure 2:**
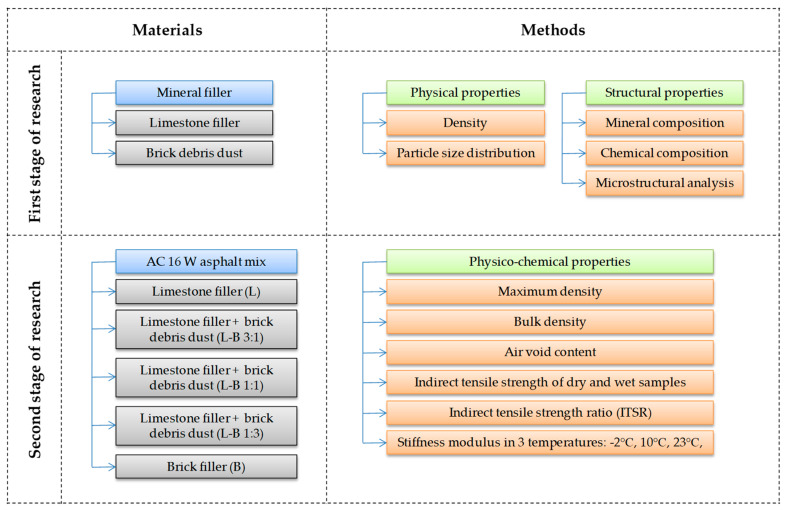
Methodology scheme used for this work.

The tests were carried out for the asphalt mix containing limestone powder as a mineral filler (reference sample) as well as the mixes containing a mixture of limestone and brick filler. Figure 2 presents the composition of the particular mixes with the proportions of materials used and summarizes the methodology applied for this study.

## 3. Research Method

### 3.1. Characterization of Lime Filler and Brick Debris Dust

The chemical analyses were performed on air-dried samples using the energy dispersive X-ray fluorescence method (EDXRF) employing an Epsilon 3 spectrometer applying the following parameters: RTG Rh 9 W, 50 kV and 1 mA lamp (Panalytical, The Netherlands). Elements within the range from Na to Am were included in the analyses.

Particle size distribution was determined using the laser diffraction method (Mastersizer 3000 device with Hydro G dispersion unit with the range of 0.02 μm to 2 mm) with the application of Mie theory and the following parameters: light refractive index of 1.52 and absorption coefficient of 0.1. The measurements were carried out with the speed of 1750 and 700 rpm for the pump and stirrer, respectively.

The phase composition of tested fillers was determined by the X-ray diffraction method (XRD) using an X’pert PROMPD diffractometer along with PW 3050/60 goniometer, Cu tube and graphite monochromator. The test angle (2*θ*) varied from 5° to 65°. Interstitial spacing parameter d_hkl_ for a particular crystal structure was identified in line with Bragg’s law.

The microstructural analysis of the fillers was performed using a Quanta 250 FEG scanning electron microscope (SEM) by FEI (Hillsboro, OR, USA).

### 3.2. Methodology of the Asphalt Mix Tests

The samples were prepared in a Marshall device and had the following dimensions: 63.5 mm height and 101.6 mm diameter. Before preparation of the first sample, the mould was heated to the compaction temperature. The compaction temperature of the reference mix asphalt was 140 °C based on the type of asphalt used. The samples for air voids and stiffness modulus test were prepared with 75 blows per each side. The samples for tests of resistance to indirect tensile strength resistance to water and frost measured by the ITSR (indirect tensile strength ratio) indicator were compacted using 50 blows per side. Samples were named with the following symbols: L for reference mix containing only lime filler, L-B 3:1 (meaning 3:1 ratio of lime to brick filler); L-B 1:1; L-B 1:3 for mixes with combined fillers and B for mix with brick filler.

The maximum density of the mix asphalt has been determined by means of the volumetric procedure according to the EN 12697-5:2012 standard.

For bulk density of the mix asphalt specimens, the ‘saturated surface-dry’ (‘SSD’) procedure was applied in accordance with the EN 12697-5: 2012 standard.

The air voids content in compacted samples was calculated based on the EN 12697-8:2005 standard.

The water and frost resistance tests of the concrete asphalts were performed based on the EN 12697-12:2008 standard and Polish technical requirements [30]. This test evaluates the effect of a single freezing cycle of saturated mix asphalt samples on indirect resistance to Marshall-type sample stretching. Ten samples were prepared with every asphalt mix type and divided into two groups: ‘dry set’ and ‘wet set’ of similar heights and bulk density values. The control series samples were conditioned in a laboratory on a flat surface. The second group of samples was exposed to a prolonged effect of water at an increased temperature by placing them in a water bath with the temperature of 40 °C for 68 h. Then, the samples were frozen for 16 h at a temperature of −18 °C and thawed for 24 h in water at a temperature of 25 °C. The samples from both sets were used to test the resistance to indirect tensile strength in the Marshall’s device, according to the EN 12697-23:2009 standard.

The ITSR indicator of resistance to water and frost was calculated based on the obtained results according to the following formula:

(1) $$ITSR=\frac{ITS_{w}}{ITS_{d}}$$

where:

ITSR—indirect tensile strength ratio [%]
ITS_w_—the average indirect tensile strength for the wet samples [kPa]
ITS_d_—the average indirect tensile strength for the dry samples [kPa]
The study of the stiffness modulus was performed according to the EN 12697-26:2012 standard.

The Marshall samples were subject to five-time dynamic load applied to the sample vertically, along the diameter. Maximum force generated a horizontal dislocation of sample equal to 5 μm. The test result was calculated automatically by a control program, as an arithmetic mean from the stiffness modulus for each of 5 force impulse measurements. The sample, after performing the test, was rotated by 90° around the horizontal axis and tested again. A reliable stiffness modulus for each sample was a mean average out of two measurements.

## 4. Results and Discussion

### 4.1. Characteristics of Brick Debris Dust and Lime Filler

#### 4.1.1. Chemical Composition

In the chemical composition of the brick debris, silicon oxide dominates, which is accompanied by a smaller amount of aluminium oxide. Moreover, the presence of MgO, CaO and K_2_O was detected in a level of several percent (Table 4). The main component of the lime filler is calcium oxide.

#### 4.1.2. Mineral Composition

**Figure 3:**
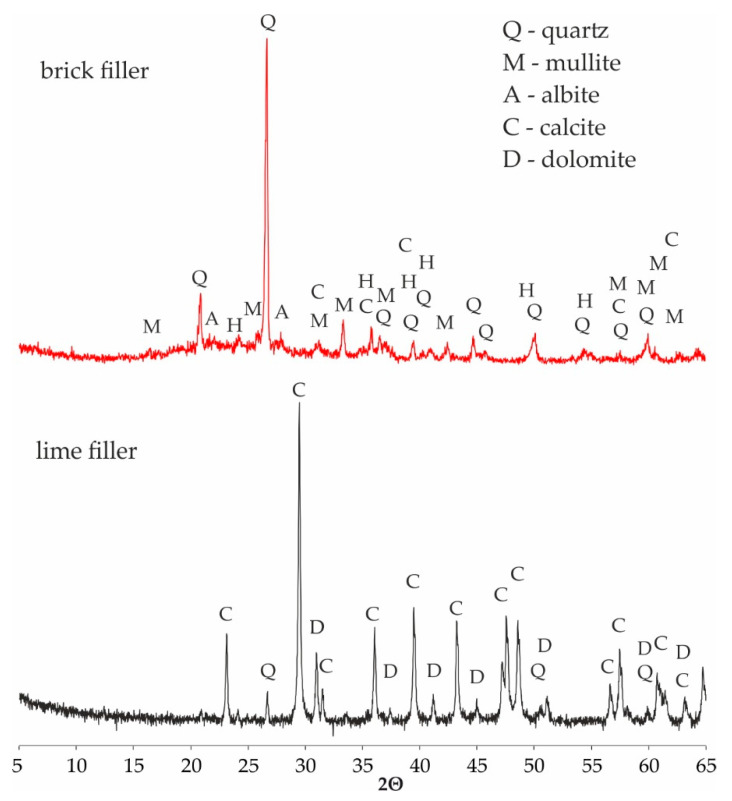
X-ray diffraction (XRD) patterns of the tested fillers.

The mineral composition of the investigated fillers is shown in Figure 3. The main mineral component of the brick debris dust is quartz which can be recognized by the characteristic d_hkl_ = 3.34, 4.26, 1.81, 2.45 Å (26.65, 20.87, 50.16, 36.56, 2θ respectively), accompanied by mullite phase of d_hkl_ = 3.39, 3.42, 5.38, 2.21 Å (26.26, 25.98, 16.44, 40.86, 2θ respectively). Another significant phase in the brick debris composition is aluminosilicate glass which can be recognized by the rise of a background line in the range 15–35° 2θ. The other, minor components are albite of d_hkl_ = 3.21, 4.03, 3.75 Å (27.73, 22.05, 23.67, 2θ respectively), hematite d_hkl_ = 2.70, 2.51, 1,84 Å (33.11, 35.61, 49.41, 2θ, respectively), and secondary crystallized calcite of d_hkl_ = 3.04, 2.07, 2.28 Å (29.40, 43.16, 39.42, 2θ, respectively). The mineral composition of the lime filler is less complex. Apart from the calcite phase which dominates, a small amount of quartz can be recognized, as well as trace amount of dolomite of d_hkl_ = 2.89, 2.19, 2.01 Å (30.97, 41.16, 44.96, 2θ, respectively).

#### 4.1.3. Scanning Electron Microscopy (SEM)

**Figure 4:**
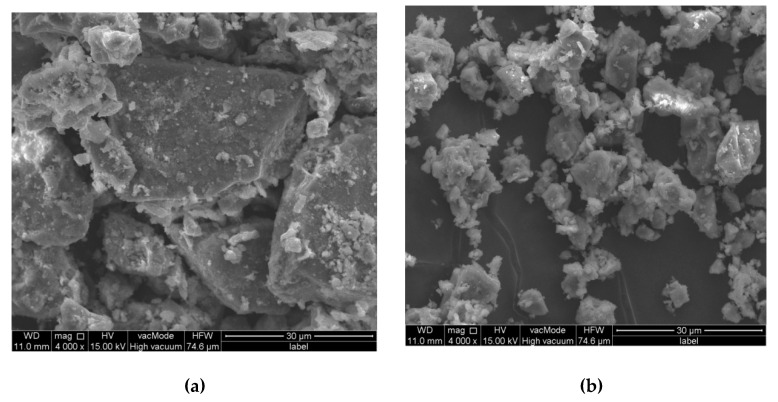
Scanning electron microscopy images of (**a**) brick debris dust and (**b**) lime filler.

The microstructural morphology of the brick debris dust and the lime filler is presented in Figure 4a,b, respectively. SEM images indicate that more fine grains are present in the lime filler. The grains in the lime filler are in the size of about 10 µm and their shape is irregular. Interestingly, the brick debris is characterized by the grains often exceeding 30 µm and their surface is heterogeneous, rough and the shape is sharp-edged in comparison to the lime filler.

#### 4.1.4. Grading

**Figure 5:**
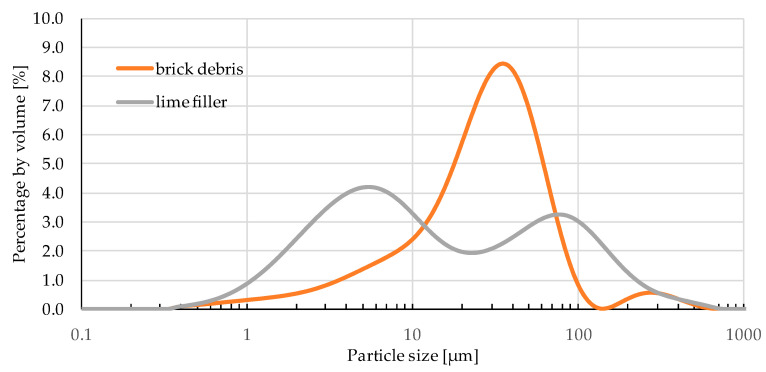
Grading curves of the tested fillers.

Figure 5 presents the particle size distribution (PSD) of two fillers used in the study. Particle size distribution of the brick debris dust is bimodal with one clear dominant peak with a maximum at about 40 µm which means that grains of this size make the greatest contribution. The second peak is relatively small with a maximum at around 120 µm suggesting a small amount of larger grains in the brick debris. Particle size distribution of the lime filler is also bimodal, however two distinct peaks can be distinguished. The first one is slightly higher and has its maximum at the value of 5 µm, and the second one at around 80 µm. The PSD curve reveals that the granulation of the lime filler is less homogeneous that granulation of the brick debris dust.

### 4.2. Properties of Asphalt Mix

#### 4.2.1. Effect of Brick Debris Dust Addition on Volumetric Properties of Asphalt Mix

**Figure 6:**
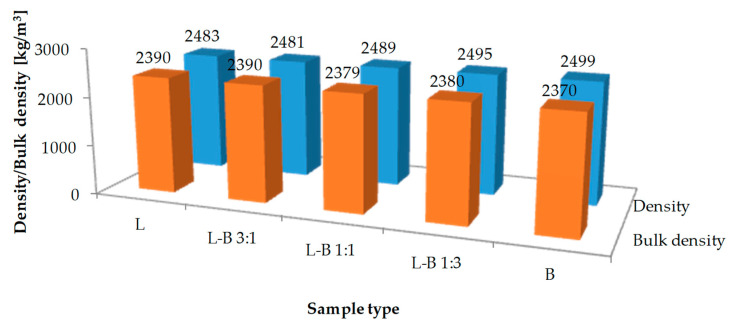
Maximum density and bulk density results of the studied mixes.

**Figure 7:**
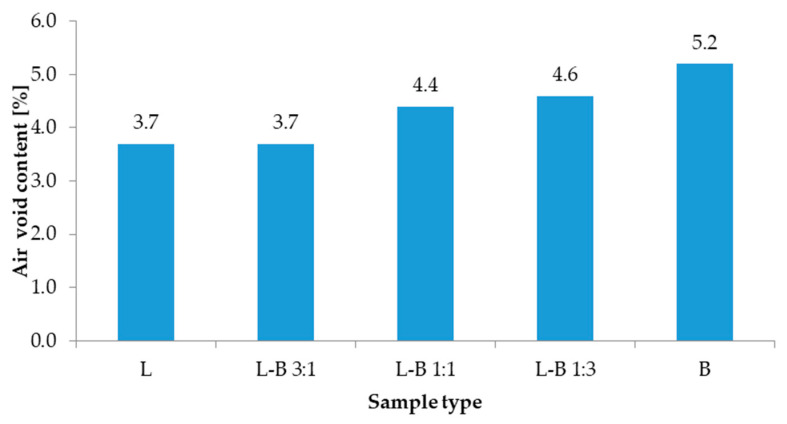
Air void content of the studied mixes.

The results of volumetric properties tests are presented in Figure 6 and Figure 7. Mean value of the maximum density of the reference mix L was 2483 kg/m^3^, while the bulk density was 2390 kg/m^3^. The value of maximum density increased from 2481 kg/m^3^ to 2499 kg/m^3^ along with a rising amount of brick dust. In the samples in which the traditional filler was replaced by 25% brick debris, the volumetric properties were comparable to those of the reference samples. Increasing the share of brick dust resulted in a steep reduction of bulk density to 2380 kg/m^3^ (50% and 75% of brick filler) and further to 2370 kg/m^3^ (100% brick filler). Also, the content of air voids increased to 4.4%, 4.6% and 5.2%, respectively, while in both reference sample L and L-B 3:1 sample the value equaled 3.7%.

Considering the comparable density of the lime filler and brick dust, minor changes in the maximum density values were expected. The trend observed in the research is different from that expected, probably as a result of the chemical composition of brick dust with a very high SiO_2_ content (76.19%), which negatively affects the properties of the obtained asphalt mastic and in particular the adhesion to the aggregate. Subsequently, the density of asphalt mix decreases, which is reflected in the bulk density and air void content values of asphalt mixes [31]. Also the particle shape of the filler has a significant impact on asphalt mix compaction process [32,33]. Particles of the lime filler used in the study are finer and have a more regular shape than those of brick dust (Figure 4). It could be seen from the SEM results that the surface of recycled brick powder is much rougher. What is more, its particle size distribution (Figure 5) is more homogeneous than that of the limestone powder. Both observations suggest that the recycled brick powder may exhibit better adsorption abilities than the limestone filler. As indicated by Zulkati et al., the filler with sufficiently large diameter and regular shape grains acts as both lubricating and fractioning agent, which facilitates the movement of the aggregates and consequently increases the compaction susceptibility of asphalt mix [33]. However, the surface of brick debris dust is more rough, which results in greater adsorption capacity than a lime filler [34]. The methodology of sample preparation is also important. According to the technical regulations in force in Poland, all samples were compacted at 140 °C, whereas Wu et al. indicate that the average compaction temperature of an asphalt mix containing brick dust should be 6.5 °C higher than that of mixes containing lime filler [25].

#### 4.2.2. Effect of Brick Debris Dust Addition on Water and Frost Resistance and Indirect Tensile Strength of Asphalt Mixtures

**Figure 8:**
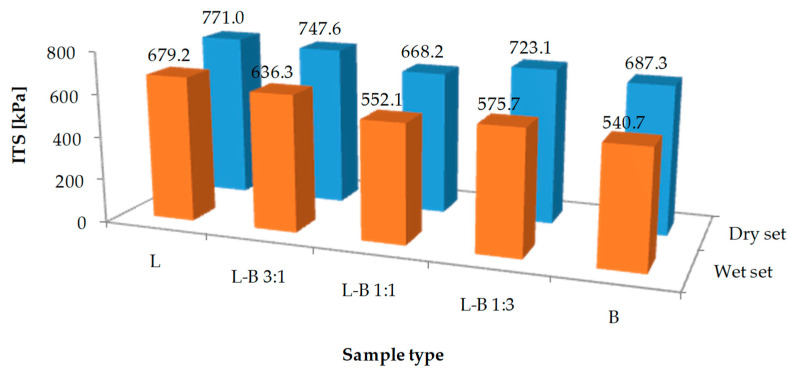
Indirect tensile strength of the studied mixes for the dry and wet samples.

**Figure 9:**
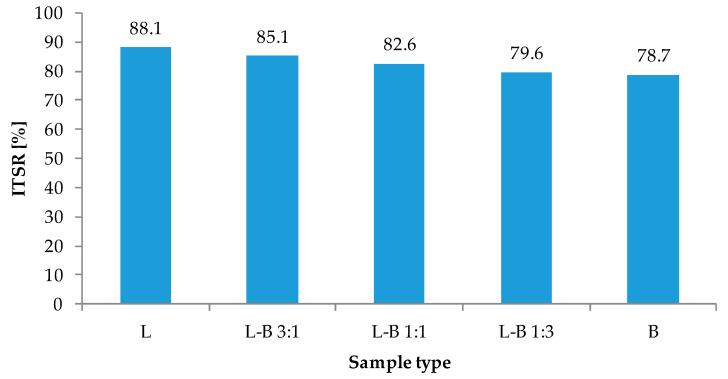
Water and frost resistance of studied mixes measured with indirect tensile strength ratio (ITSR) indicator.

The results of the strength properties test and resistance to water and frost are shown in Figure 8 and Figure 9. The reference asphalt mix L had the highest indirect tensile strength values of 770.1 MPa for dry samples and 679.2 MPa for wet samples, respectively. The decrease in strength as a result of water and frost was the lowest in samples with lime filler, so the ITSR index reached 88.1%. The use of dust from brick debris as a filler resulted in a reduction in the strength of the produced mixture as well as lower water and frost resistance. Samples in which the lime filler was half replaced by brick filler (L-B 1:1) were the weakest in terms of strength (dry samples—668.2 MPa). Taking into account the resistance to water and frost, a decrease in the ITSR parameter from 85.1% to 78.7% was observed with the increase in the share of brick dust in the filler.

One of the key factors influencing water and frost resistance is the adhesion of asphalt to aggregates, including fillers. Bonding of the asphalt mix components to a large extent depends on the chemical and mineral composition of fillers. Materials that exhibit hydrophobic properties adhere very effectively to the asphalt. In turn, natural aggregates used in road construction are hydrophilic to varying degrees (water attracting), but also lipophilic (fat attracting). Asphalt binder has a hydrophobic character and, therefore, if the applied filler shows more affinity to water than to bitumen (high hydrophilicity), the asphalt mastic produced will not be durable. In addition, the created joints will be additionally weakened by water during the operation of the pavement.

As noted by Nciri et al., a filler with with a high calcium content is naturally a material which forms strong bonds in contact with hydrophobic organic compound (i.e., asphalt binder) [15]. This theory can explain the higher tensile strength values of asphalt mixes with calcium filler in which calcium carbonate (Table 4) occurs in the form of calcite (Figure 3). In contrast, the chemical composition of brick dust is mainly SiO_2_ (Table 4) in the form of quartz (Figure 3). Moreover, the surface of silica materials is characterized by the occurrence of hydroxyl groups providing hydrophilic character. Both the acidity of this material and its hydrophilic character adversely affect the asphalt adhesion to the aggregate, which is reflected in a decrease in tensile strength and resistance to water and frost.

A decrease in water and frost resistance can lead to premature degradation of the road surface. However, the use of brick dust is an interesting recycling method. Therefore, it is necessary to determine the optimal amount of brick dust in the mixed filler. Considering the requirements of ITSR for the binding layer, (at least 80%) it is possible to replace up to 50% of the lime filler with brick debris dust (Figure 9).

#### 4.2.3. Effect of Brick Debris Dust Addition on Stiffness Modulus of Asphalt Mixtures

**Figure 10:**
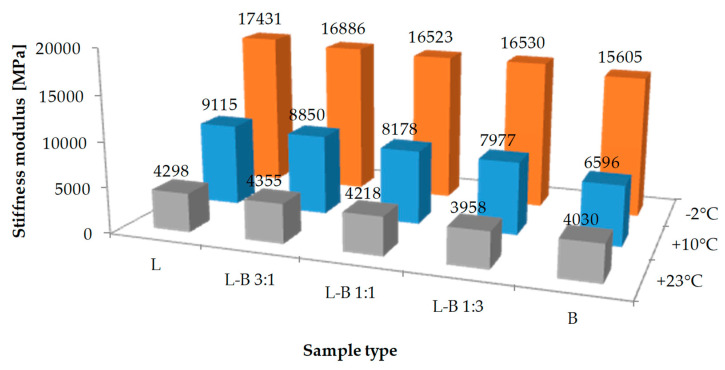
Average stiffness modulus values of studied mixes.

The mean values of stiffness modulus are presented in Figure 10. Reference sample L had the highest value of stiffness modulus at both subzero (−2 °C) and average temperatures (+10 °C). Below 0 °C, the decrease of stiffness modulus was observed for samples containing brick filler. The values were from 16,886 MPa for L-B 3:1 samples to 15,605 MPa for B samples. Considering the fact that road surfaces become stiffer over time [35], the results obtained should be treated positively in terms of asphalt mix low-temperature cracking resistance.

The stiffness of reference samples (L) at a temperature of +23 °C was 4298 MPa. The replacement of the lime filler with brick debris dust caused a change in stiffness at that temperature ranging from +1.3% to −7.9%. However, for samples where the lime filler has been replaced by brick dust by no more than 50%, the stiffness remains close to the level of reference samples. Due to the fact that asphalt mixes of high stiffness modulus at positive temperatures are more resistant to permanent deformations [36], it can be concluded that adding more than 50% of brick dust into the filler is more likely to cause rutting in the road surface. Based on the equivalent temperature of softening and cracking of asphalt material, Wu et al. found that the advantage of asphalt mastic containing brick dust is its resistance to high temperatures and the disadvantage is low-temperature properties [25]. Due to divergent conclusions resulting from the tests of asphalt mastics described in the literature and the analysis of the results obtained, these properties should be examined according to the procedures in force in this field.

The analyses mentioned above are based on generally known probable correlations described in the literature. Nonetheless, it is worth noting that a material with originally low mechanical properties can stiffen faster than a material with high initial stiffness. The stiffness of mineral–asphalt mixtures and its change over time is mainly influenced by the type of asphalt, including chemical composition and molecular structure. However, as indicated by Gandhi et al., even when the same type of asphalt was applied, the change of stiffness for the aged samples was different for various aggregates and other warm mix asphalt additives [37].

As expected, the stiffness of asphalt mix with the addition of brick dust decreased with the increase of voids in the samples [35,38]. Asphalt changes its properties over time due to oxidation processes. In layers of asphalt pavements with a higher air void content, the oxidation of the asphalt binder is expected to be more intense. In consequence, these mixtures, despite their lower initial stiffness, can stiffen faster.

### 4.3. Statistical Data Analysis

The one-way analysis of variance (ANOVA) method was implemented for statistical analysis of particular test group results. This parametric tool allows the comparison of more than two groups separated by the categories of one variable being an extension of the *t*-test used to compare two independent groups. The results of the ANOVA analysis are presented in Table 5. The *p* values indicate that the results are statistically significant in every test (*p* < 0.05). Moreover, to indicate the replicability of the particular tests, in Table 6 standard deviation values for each group were presented.

## 5. Conclusions

The paper presents the influence of brick debris dust addition on the properties of asphalt mix. The use of mixed lime-brick filler could contribute to the increase in recycling of brick debris obtained from the demolition of old buildings and structures. On the basis of the experimental work conducted, the following conclusions can be drawn:

Along with the increase in the quantity of brick filler, a decrease in the bulk density was generally observed, which may be the result of irregular-shaped grains of brick, and thus reduced compaction susceptibility. Replacement of the lime filler with brick dust in the amount of 25% did not affect the volumetric properties of asphalt mix, specifically maximum density, bulk density and air void content. Further increase in the percentage of brick dust caused a decrease in bulk density and increase in maximum density as well as air voids. Both the chemical character and the mineral composition determine the strength of the bitumen-filler bond. A high proportion of silica in the brick, which has an acidic and hydrophilic chemical character, is associated with a reduction in the binding force between the filler and binder. Therefore, the addition of brick dust filler resulted in a decreased tensile strength of asphalt mix along with lower resistance to water and frost. However, the ITSR value required by technical regulations was maintained in mixtures with mixed filler containing up to 50% brick dust.

The results of the stiffness modulus tests of asphalt mix suggest that the addition of brick dust will have a positive effect on the resistance to low-temperature cracking. At the same time the resistance to permanent deformation should not change if the amount of brick dust in the filler does not exceed 50%.

The analysis of the examined physico-mechanical properties of asphalt mix indicates that brick debris dust can be used as a material in the mixed filler, while its percentage should not exceed 50%. Both the acidity of brick dust and its hydrophilic nature in regard to water have a negative impact on the adhesion to the asphalt, which is reflected in the properties of the mineral–asphalt mixture and, as a consequence, reduced service life of the road surface. Conversely, parameters such as homogeneous filler gradation and rough grain surface ensure good adhesion between the binder and the filler, which to some extent may compensate for the negative effect of the chemical nature of the brick filler surface on the asphalt mix properties. Application of an appropriate ratio of dust from brick debris allows the design of an asphalt mix with good performance parameters. The proposed solution fits the idea of sustainable development, indicating a means of brick debris management.

## Figures and Tables

**Table 1:** Physical properties of lime filler and brick debris dust used in the tests.

Test	Specification	Result	
		Lime Filler	Brick Debris Dust
Grain size [mm]	EN (European Standard) 933-10:2009	0–2	0–2
Density [kg/m^3^]	EN 1097-7:2008	2763	2782
Water content [%]	EN 1097-5:2008	<1	<1

**Table 2:** Properties of the bitumen.

Test	Specification	Result	Specification Limits
Penetration (25 °C; 0.1 mm)	EN (European Standard) 1426:2009	38.2	35–50
Softening point (°C)	EN 1427:2009	55.8	50–58
Viscosity at 135 °C (Pa·s)	ASTM (American Society for Testing and Materials) D 4402	0.755	-
Penetration index	EN 12591:2010	−0.47	-

**Table 3:** Composition of mineral mixture and asphalt mix.

Type of Aggregate	Grain Size [mm]	Origin	Composition [m%]	
			Mineral Mixture	Asphalt Mix
Dolomite 0/4	0–4	Łagów, Poland	24.5	23.4
Dolomite 2/8	2–8	Łagów, Poland	37	35.3
Dolomite 8/16	8–16	Łagów, Poland	34	32.5
Limestone filler	0–2	Bukowa mine, Poland	4.5	4.3
35/50 asphalt		ORLEN Asfalt Sp. z o.o		4.5
Total			100	100

**Table 4:** Chemical composition of the tested fillers.

	Brick Debris	Lime Filler
[% of Weight]		
MgO	1.29	0.21
Al_2_O_3_	9.68	0.15
SiO_2_	76.19	0.27
P_2_O_5_	0.00	0.02
SO_3_	0.04	0.04
K_2_O	3.00	0.00
CaO	4.54	74.68
TiO_2_	0.84	0.00
Fe_2_O_3_	4.13	0.06

**Table 5:** Analysis of variance (ANOVA) analysis on the particular tests.

Test Type	Origin	SS	df	MS	F	*p*	Test F
Density	Intergroups	721	4	180	80	1.54 × 10^−7^	3.478
	Intragroups	23	10	2			
Bulk density	Intergroups	837	4	209	18	1.34 × 10^−4^	3.478
	Intragroups	114	10	11.4			
Indirect tensile strength (wet)	Intergroups	55,728	4	13,932	8.657	0.0008	3.056
	Intragroups	24,140	15	1609			
Indirect tensile strength (dry)	Intergroups	28,469	4	7117	6.141	0.0039	3.056
	Intragroups	17,385	15	1159			
Stiffness modulus at 23 °C	Intergroups	705,714	4	176,428	3.103	0.0333	2.759
	Intragroups	1,421,308	25	56,852			
Stiffness modulus at 10 °C	Intergroups	23,202,499	4	5,800,625	17.806	4.94 × 10^−7^	2.759
	Intragroups	8,144,274	25	325,771			
Stiffness modulus at −2 °C	Intergroups	10,638,660	4	2,659,665	4.344	0.0084	2.759
	Intragroups	15,307,063	25	612,283			

SS: Sum of the squared deviations; df: Degree of freedom; MS: Mean square; F: F-value; *p*: *p*-value. Test F: Test F-value.

**Table 6:** Standard deviation values for particular groups of samples.

	Standard Deviation Values				
Test	L	L-B 3:1	L-B 1:1	L-B 1:3	B
Density	1.2	1.2	2.1	0.6	2.0
Bulk density	3.0	3.2	2.5	2.5	5.0
ITSw	42	60	34	18	34
ITSd	25	34	27	38	44
Stiffness at 23 °C	218	329	211	139	254
Stiffness at 10 °C	585	429	763	262	672
Stiffness at −2 °C	385	1072	1258	299	306

ITSw: Indirect tensile strength for wet samples; ITSd: Indirect tensile strength for dry samples.
